# Prioritizing reducing impacts on species to minimize residual reserves

**DOI:** 10.1126/sciadv.aeh8129

**Published:** 2026-10-21

**Authors:** Aharon G. Fleury, Casey C. O’Hara, Jason D. Everett, Benjamin S. Halpern, Carissa J. Klein, Anthony J. Richardson, Daniel C. Dunn

**Affiliations:** ^1^ Centre for Biodiversity and Conservation Science, The School of the Environment, The University of Queensland, St. Lucia 4072, Queensland, Australia.; ^2^ National Center for Ecological Analysis and Synthesis, University of California–Santa Barbara, Santa Barbara, CA 93101, USA.; ^3^ Centre for Marine Science and Innovation, The University of New South Wales, Sydney 2052, New South Wales, Australia.; ^4^ Bren School of Environmental Science and Management, University of California–Santa Barbara, Santa Barbara, CA 93106, USA.

## Abstract

International conservation goals have driven rapid expansion of protected areas. This growth has favored quantity over quality, and often results in residual reserves (i.e., areas where species already experience minimal anthropogenic impact). Spatial prioritization is commonly used to help design protected area networks that represent species while minimizing costs to stakeholders. This approach can result in the siting of protected areas that do not decrease impacts on species of conservation concern. Here, we show how measures of cumulative impacts on species can be used in a spatial prioritization to site protected areas to reduce impacts on species of conservation concern. We use a trait-based vulnerability framework and anthropogenic stressor maps to quantify cumulative impacts from four human activities, and incorporate those impacts into spatial prioritizations. We demonstrate that the inclusion of cumulative impacts on species in spatial prioritization can more efficiently protect biodiversity, balance socioeconomic objectives, and avoid residual reserve outcomes.

## INTRODUCTION

Human activities, such as transport, resource extraction, and infrastructure development, continue to intensify around the world, resulting in increased impacts on ecosystems (*1*–*6*). In response, international commitments have advocated for the sustainable use of all ecosystems and protection of 30% of the land and ocean by 2030 (*7*, *8*), contributing to rapid growth in the designation and size of protected areas (*9*, *10*). This strategic growth in protected areas needs to be based on a diverse range of management objectives. Systematic conservation planning often deliberately prioritizes low-impact areas to achieve broad representation of intact ecosystems (*11*–*13*), secure regional connectivity (*14*), or establish replication that hedges against future climate anomalies (*15*). However, when the designation process is driven primarily by the need to minimize socioeconomic conflict or meet rapid area-based targets, it can inadvertently bias protection toward low-risk zones at the expense of high-impact areas. Consequently, this can result in the creation of “residual reserves” (*16*–*26*)—protection configurations that, while ecologically valuable in isolation, fail to displace active human impacts or optimally contribute to broader biodiversity persistence (*27*).

Residual reserves manifest in part from the push to meet area-based international commitments (*28*). These commitments, commonly set with short deadlines, often result in governments favoring simpler, lower-conflict approaches to meeting area-based targets, including the designation of fewer, larger protected areas in lower-use areas, such as overseas territories (*17*). For example, the 2022 Kunming-Montreal Global Biodiversity Framework gives governments less than a decade to substantially expand protected areas, creating strong incentives for rapid, easily implemented approaches (*7*). This focus has encouraged expansion over effectiveness, or quantity over quality, leading to shortfalls in biodiversity representation and threat mitigation (*29*–*32*). These reserves also arise from the methodological approaches used to achieve those commitments. Spatial prioritization offers a structured approach for identifying cost-effective solutions for achieving conservation objectives (*33*, *34*). Conservation planning problems are commonly configured to minimize costs while meeting area-based targets for biodiversity features, such as species distributions, because conservation funds are often limited and planning therefore requires the effective and efficient use of available resources (*35*). By minimizing costs, these outcomes typically avoid areas with high levels of human activity, and hence divert conservation away from places where biodiversity may face the greatest human impacts.

An additional problem is that typical problem formulations assume that impacts on a species are spatially homogeneous across its distribution. This assumption fundamentally limits the ability of spatial prioritizations to guide actions that directly reduce impacts on species. The resulting outcomes tend to prioritize protected areas that avoid conflict with stakeholders over protecting affected species (*18*, *36*–*38*). Assuming homogeneous impacts on a species tends to drive protection toward low-impact areas and the misallocation of limited conservation resources (*39*). Integrating impacts into spatial prioritizations could enhance conservation efforts by targeting areas where species are most vulnerable to and affected by anthropogenic threats (*21*).

Impacts vary widely among species because differences in ecological and life-history traits, such as size, mobility, or habitat use, mediate how species respond to stressors (*40*–*44*). Trait-based approaches offer a way to capture these differences by extrapolating complex processes to generic descriptors (*45*, *46*), providing a scalable method to assess thousands of species without bespoke information on individual species. As such, global trait-based frameworks have been developed to quantify species’ vulnerability to anthropogenic stressors, with a particular focus on marine taxa (*47*, *48*). Among these frameworks, the one developed by Butt *et al.* (*47*) represents the most extensive global vulnerability assessment for marine species.

Their framework evaluates adaptability and sensitivity (i.e., vulnerability) of a marine species to multiple threats on a global scale. They applied this trait-based framework to estimate the vulnerability of >44,000 marine vertebrate and invertebrate species to 22 anthropogenic stressors, using a total of 42 traits from five categories (movement, reproduction, specialization, spatial scale, and biophysical traits). The framework was then expanded by incorporating stressor intensities to calculate the impact of 13 anthropogenic stressors on 21,267 marine species (*6*). The scale of this framework enables conservation objectives focused on reducing impact to species rather than meeting area-based biodiversity targets.

Here, we present an approach to reduce residual reserve outcomes in spatial prioritization by reframing the conservation problem to prioritize the reduction of impacts on species. We use a global analysis with 773 vulnerable, endangered, and critically endangered marine species and four anthropogenic stressors that can be mitigated with marine reserves: fishing, bycatch, shipping, and habitat loss or degradation. Cumulative impact for each species is quantified assuming the additive effect of all four anthropogenic stressors on each species, using the trait-based species vulnerability framework (*47*) together with stressor intensities from O’Hara *et al.* (*6*). We then implement three different prioritization scenarios: a Reference scenario that prioritizes species representation (i.e., the common prioritization approach), an Impact scenario that prioritizes impact reduction on species, and a Hybrid scenario that prioritizes both species representation and impact reduction. We explore how incorporating impacts into spatial prioritization redistributes protected areas and influences the efficiency of impact reduction relative to cost and area. This framework demonstrates how trait-based species vulnerability can be incorporated as impacts in spatial prioritizations to reduce residual reserve outcomes on both land and sea, and efficiently strengthen protection of species and allocation of conservation funds.

## RESULTS

We used the minimum set objective (ensures all targets are met while minimizing the cost of the solution) for each of the three prioritization setups (Table 1). The Reference scenario used 773 features (the species’ distributions). The Impact scenario uses 773 features, which represents the cumulative impact of all four stressors on each species within their distribution. The Hybrid scenario uses all the features from both the Reference and Impact scenarios. For the minimum set objective, targets are provided for each of the features. For species distribution features, we used the 30% by 2030 target (i.e., 0.3). For impact distribution features, we calculated the total cumulative impact reduction (CIR) percentage summed across all species from the Reference scenario solution and implemented that value as the impact targets for each species in the two other scenarios. Spatial variation and efficiencies of the Impact and Hybrid scenarios were compared against the Reference scenario.

**Table 1.** Prioritization problem setup for each scenario in prioritizr. NA, not applicable to this scenario; CIR, cumulative impact reduction.

Scenario	Objective	Features	Number of features	Species distribution target	Cumulative impact target	Cost (US$/PU)	Gap
Reference (species features)	Minimum set	Species distributions	773	30%	NA	Commercial fishing value	0.01
Impact (impact features)	Minimum set	Cumulative impact per species	773	NA	Reference CIR value: 0.173	Commercial fishing value	0.01
Hybrid (species and impact features)	Minimum set	Species distributions, cumulative impact per species	1546	30%	Reference CIR value: 0.173	Commercial fishing value	0.01

### Spatial redistribution of protection with incorporation of impacts

The spatial concordance of the Reference scenario with the Impact (Fig. 1A) and Hybrid scenarios (Fig. 1B), measured with Cohen’s kappa (κ) (*49*), showed only “fair” agreement (κ = 0.34). By contrast, there was “almost perfect” agreement (κ = 0.90) between the Reference and Hybrid scenarios (*50*). These results indicate that prioritizations based solely on cumulative impacts produced substantially different spatial solutions relative to the Reference species–only scenario.

**Fig. 1.**
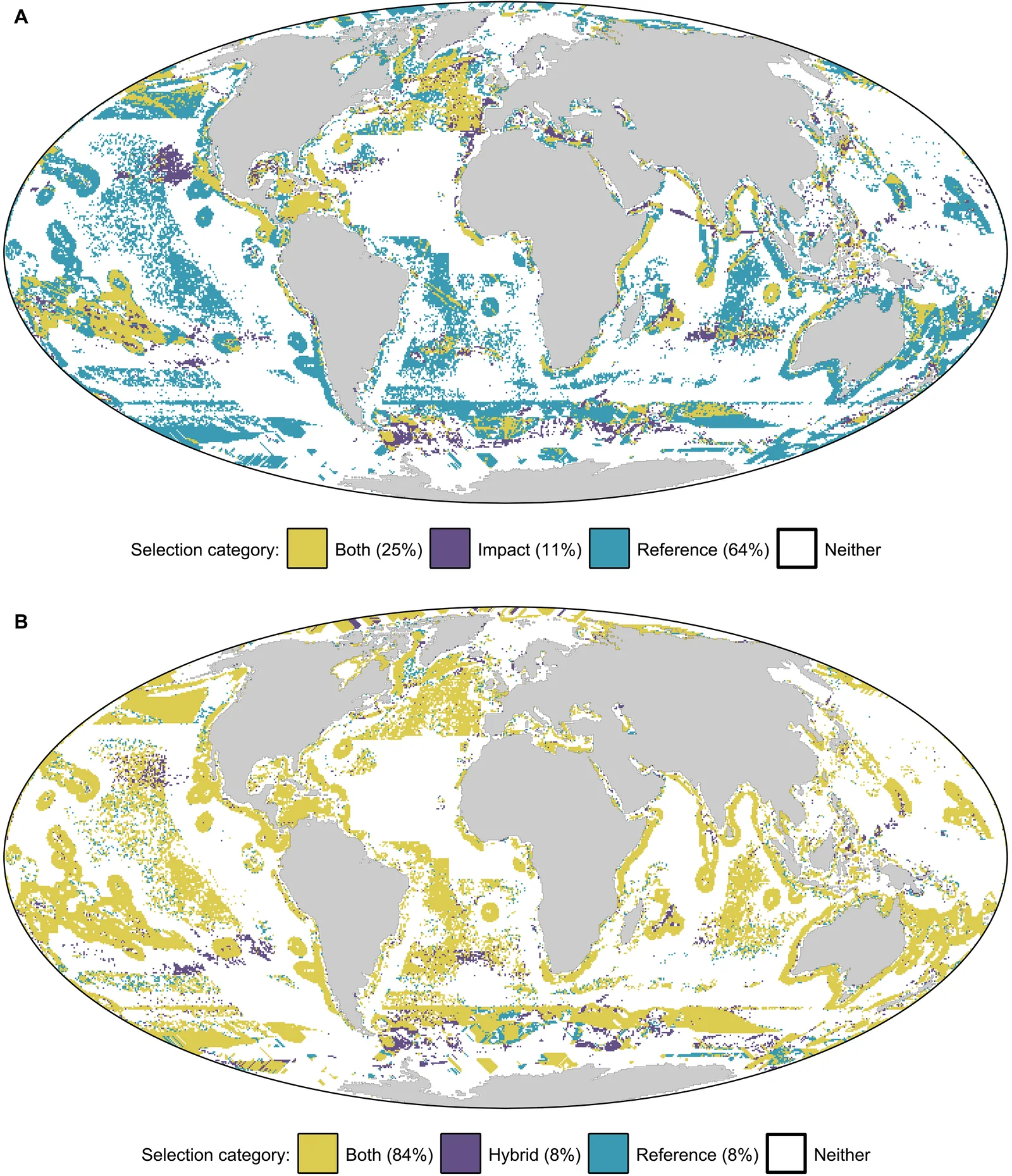
Comparison of spatial variation of priority areas between planning scenarios. Maps show planning unit selection for each scenario relative to the Reference scenario. Colors denote how the two scenarios selected planning units: both scenarios (gold), alternative scenario (purple), reference (blue), or neither (white). Percentages represent the proportion of selected planning units in each category excluding the “Neither” category. (**A**) Impact scenario compared with the Reference scenario. (**B**) Hybrid scenario compared with the Reference scenario.

Overlapping planning units (PUs) accounted for a larger proportion of the total selected PUs for the Impact scenario (69%) than in the Reference scenario (28%). This difference primarily reflects the ability of the Impact scenario to meet impact-only targets using substantially fewer PUs (17,660) than the Reference scenario required to meet species targets (43,826). This pattern aligns with the expectation that impacts are spatially more concentrated than species distributions, and therefore require less area to achieve equivalent target levels. In contrast, the Hybrid and Reference scenarios shared an equal proportion of overlapping PUs (91%) with both selecting a similar total number of PUs (44,115 for the Hybrid scenario).

Although the primary aim of the analysis was to demonstrate an impact-reduction framework, there are some notable regions selected that are interesting. Areas selected by the Impact and Hybrid scenarios, but not by the Reference scenario, were primarily associated with high fishing and bycatch impacts (Fig. 1, A and B). For all scenarios, selected areas in several regions, including the north Atlantic and Caribbean, were driven by shipping impacts. The fine-scale fragmentation observed across scenarios likely reflects the absence of a boundary length modifier in the planning problems. In contrast, larger block-like artifacts likely reflect the structure of the fisheries cost layer, where catch and revenue estimates derived partly from coarse Food and Agriculture Organization fishing regions introduced region-level patterns and abrupt spatial transitions in estimated opportunity costs. These large artifacts may be reduced using finer-resolution fisheries datasets, such as national fisheries data, for the cost layer.

### Comparison of impact efficiencies

Summary statistics for each scenario, including the efficiency of the CIR by area (% per 10^7^ km^2^) and cost (% per billion US$), are provided in Table 2. These efficiency metrics are used to evaluate how effectively each scenario reduces residual reserves by achieving greater CIR per unit area and cost.

**Table 2.** Summary of prioritization scenario results. PUs, planning units; CIR, cumulative impact reduction; NA, not applicable to this scenario.

Scenario	Species distribution target	Cumulative impact target	Number of planning units (out of a total of 145,894 PUs)	Total cost (billion US$)	Total cumulative impact reduction (%)	Impact cost efficiency (CIR % per billion US$)	Impact area efficiency (CIR % per 10^7^ km^2^)
Reference	30%	NA	43,826	10.9	17.3	1.58	1.58
Impact	NA	0.173	17,660	6.52	19.6	3.01	4.45
Hybrid	30%	0.173	44,115	11.7	21.0	1.80	1.90

The Impact scenario achieved a 14% increase in relative CIR compared to the Reference while meeting the same impact target derived from the Reference solution applied to each species. This improvement reflects the prioritization identifying a more optimal spatial solution when cumulative impacts are used as features rather than species distributions. The Impact scenario required 60% less area and 40% less cost than the Reference scenario to achieve this outcome, resulting in nearly double the cost efficiency and triple the area efficiency for reducing cumulative impacts.

The Hybrid scenario also demonstrated improved efficiency relative to the Reference. Compared with the Reference, the Hybrid scenario achieved a 21% relative increase in CIR, alongside a 20% relative increase in area efficiency and 14% relative increase in cost efficiency for reducing impact. Despite meeting the same species distribution targets and using the Reference CIR as the impact target, the Hybrid scenario achieved greater CIR with less than 1% additional area and only a 7% increase in cost, indicating more efficient use of both area and cost while meeting the 30% protection targets.

## DISCUSSION

The relative ease of achieving area-based conservation targets has contributed to the proliferation of residual reserves (*17*, *18*, *22*, *25*, *26*, *28*). We demonstrate a prioritization approach for reducing such outcomes by incorporating species-specific cumulative impacts within spatial prioritization. In our example, integrating cumulative impacts while maintaining 30% ocean protection resulted in a 21% relative increase in CIR for only 1% more area and 6% higher cost. Our efforts show that the incorporation of impacts into spatial prioritizations can reduce the potential for residual reserves by more efficiently targeting areas where species are most affected while better balancing conservation needs with socioeconomic considerations.

With the push to meet global targets, such as 30% protection by 2030, and limited funds for conservation, it is necessary to identify approaches that efficiently protect species from anthropogenic threats while maintaining biodiversity (*21*). The Impact scenario was the most efficient scenario for reducing cumulative impact with regard to area and cost; however, it is probably unrealistic as it targets areas with activities that drive the blue economy. This scenario could cause both political conflict and potentially increased economic hardship for all stakeholders (*51*–*54*). A more realistic approach is the Hybrid scenario, which incorporates both species distributions and cumulative impacts as features. The Hybrid scenario remained substantially more efficient than the Reference scenario across all three metrics: CIR, area efficiency, and cost efficiency. In addition, the Hybrid scenario met the 30% protection commitment for all species targets and equitably reduced impact across all species. Thus, the Hybrid scenario offers an efficient, balanced approach to conservation and socioeconomic interests, which may lead to more realistic discussions with stakeholders and fewer residual reserve outcomes.

Our approach represents one of several possible methods for identifying and incorporating impacts into spatial prioritization to reduce residual reserve outcomes. Species vulnerability, a central component of our framework, can be quantified using a range of approaches, including correlative, mechanistic, trait-based, or combined methods (*55*, *56*). Impacts may also be incorporated into prioritization problems through alternative formulations, such as using different objectives or treating impacts as a penalty or constraint (*57*). The impact targets used in our approach emphasized equitable reductions across all species, shifting protection from minimizing aggregate impact to reducing impacts at a species level in the Impact and Hybrid scenarios. Impact targets could allow impact reduction to be unevenly distributed among species, potentially prioritizing total impact reduction over equity depending on management objectives. These alternatives highlight that multiple valid impact-based prioritization designs are possible and that different formulations may be appropriate for addressing ineffective reserves depending on the conservation context. Accordingly, while our framework provides a pathway for reducing residual reserves, it should be viewed as part of a broader toolkit for impact-informed conservation planning.

The cumulative impact data that we used represent species at a single life stage, typically as an adult, but impacts on species may change across life stages (*58*, *59*), with consequences for prioritization solutions to protect species throughout their life cycles (*60*). A species’ life history can influence prioritization outcomes as vulnerability may shift among breeding, feeding, nursery, and migratory areas (*14*, *61*). Further, many anthropogenic stressors, such as shipping and fishing, vary temporally, which can alter cumulative impact estimates over time (*62*). While our analysis evaluates cumulative impacts at a single point in time, incorporating the spatial and temporal dynamics of both species and stressors may further improve efficiency and enable more targeted impact reduction across seasonal or interannual timescales.

PU characteristics, including size and shape, can influence the efficiency of the conservation area (*63*). Because our analysis was conducted globally, the species distribution and impact datasets used here are necessarily coarser and more uncertain than datasets that may be available for well-documented regions. Species distributions and human activities often vary at much finer spatial scales; consequently, coarse PUs may mask underlying heterogeneity and constrain how precisely prioritization can target impacts. The similarities observed between the Reference and Hybrid scenarios in our analysis are therefore likely influenced, in part, by the large PUs and global datasets used. The framework may be particularly useful at regional scales or larger, where vulnerability can be incorporated across broader groups of species and stressor exposure can be represented consistently across PUs. Local applications may require finer-resolution vulnerability data to capture fine-scale heterogeneity in species responses to localized stressor intensities. Applying the framework at finer spatial scales with detailed data could yield stronger differentiation between scenarios and enable more targeted reductions in impacts.

The inclusion of different stressors in spatial prioritization should be guided not only by species vulnerability, but also by the feasibility of reducing those stressors through management actions (*64*). However, the inclusion of nonmanageable anthropogenic stressors such as climate change can still provide valuable insight into cumulative impact and conservation planning. Species’ vulnerability varies across stressors (*47*), and including all stressors may help clarify the proportion of cumulative impact that can realistically be reduced. Even when only a subset of stressors are directly manageable, reducing these stressors may still benefit species by lowering overall stress, reducing negative carry-over effects, and improving resilience to climate change (*65*–*67*).

The aim of an impact-based approach is not to exclude low-impact areas from conservation, but to reduce their preferential selection within a prioritization when they contribute little impact mitigation. Intact or lightly affected areas still hold conservation value, particularly for maintaining ecological integrity and long-term biodiversity persistence (*11*–*13*). Therefore, effective spatial planning may require integrating information on species distributions, conservation value, cumulative impacts, and the socioeconomic consequences of protection. By combining these elements, conservation planning can achieve two complementary goals: protecting wilderness areas from future degradation and mitigating existing impacts on vulnerable species.

It is important to consider how human impacts may displace following establishment of protected areas to ensure effective management (*68*), although these effects are complex and difficult to account for (*69*). Individual impacts may often displace outside of the protection area, reducing the benefits and efficiencies of the newly designated protected area (*70*). Thus, conservation designs that account for socioeconomic consequences and policy strategies that minimize stakeholder costs will be most effective (*71*). Incorporating cumulative impact into spatial prioritization can provide a more efficient solution for protecting species, but management actions will benefit from considering both the conservation and socioeconomic consequences of displaced human activities in all scenarios. While we did not account for displacement in this study, we treat each scenario similarly and assume that inclusion of displacement would decrease impact reduction across all scenarios.

Incorporating cumulative impacts into spatial prioritization represents only one step within systematic conservation planning. Effective implementation still depends on management, funding, stakeholder engagement, and the legal frameworks within which conservation policies are applied. Existing guidelines and toolboxes [e.g., Borja *et al.* (*72*)] can support decision-makers and stakeholders in identifying impact-reduction policies and actions. However, continued investment in protected areas without adequate financial support or effective enforcement can limit conservation effectiveness (*73*, *74*). Prioritizing areas with high cumulative impacts may also be less socially or politically acceptable where impact reduction creates direct trade-offs with industry revenue. Cost layers often cannot fully capture broader socioeconomic acceptability, which will depend on stakeholder priorities, compensation mechanisms, and governance context (*75*). These constraints vary across jurisdictions, meaning that the feasibility and effectiveness of impact-reduction actions will depend on the governance context in which they are implemented.

A key consideration when comparing these scenarios was ensuring that differences in outcomes reflect the impact features rather than differences in parameterization. Therefore, we deliberately used simple planning problem configurations across all three scenarios and configured the Impact and Hybrid scenario cumulative impact targets using the total CIR from the Reference solution. We did not use scenario-specific parameters, such as boundary length modifiers, as our intent was to isolate the effect of incorporating cumulative impact rather than to produce optimized protected-area designs for implementation. Future applications of the framework intended for operational conservation planning should include appropriate calibration of the planning problem to reflect stakeholder objectives and uncertainty in the data.

As anthropogenic stressors continue to intensify over the coming decades (*3*), it is more critical than ever to account for impacts to vulnerable species to reduce residual reserve outcomes. The pursuit of conservation quantity over quality is detrimental to species protection and risks perpetuating inefficiencies in conservation planning (*76*). Given limited financial resources and social capacity for conservation actions, it is essential that protected areas deliver meaningful reductions in impacts on species and habitats. By integrating cumulative impact into spatial prioritization, conservation efforts can move toward more effective protected areas that deliver greater biodiversity benefits while better balancing ecological and socioeconomic priorities.

## MATERIALS AND METHODS

We developed three spatial prioritization scenarios. The first was a Reference scenario that included only species distributions as features with associated conservation targets (e.g., protect 30% of the land and ocean by 2030), which is representative of common prioritization configurations. The other two were Impact scenarios that incorporated anthropogenic impacts on species, which we compared to the Reference species–only scenario. These were (i) an Impact scenario that used cumulative impact on species as separate features with associated targets; and (ii) a Hybrid scenario that included both species distributions and cumulative impact on species as separate features with associated targets. Our global analysis evaluated impacts from four anthropogenic stressors: targeted fishing, bycatch, shipping, and habitat loss or degradation. We limited our study to only stressors that could be directly managed by the implementation of a protected area. As a result, climate-related or indirectly manageable stressors (e.g., pollution or run-off) were not included, although marine protected areas may also be able to improve resilience to climate stressors (*67*, *77*). Spatial data were calculated on a gridded global map using a Mollweide equal-area projection (ESRI:54009) coordinate reference system with a resolution of 50 km by 50 km. This spatial resolution was selected to reduce prioritization run times when demonstrating the framework; however, the methodology and comparability of results presented here are not dependent on the resolution used.

### Species selection and distributions

For our study, we selected marine species that were listed as Vulnerable, Endangered, or Critically Endangered in the IUCN Red List version 2025-1 (*78*), which included IUCN spatial distributions, and were also included in the Butt *et al.* (*47*) trait-based vulnerability framework. The IUCN species distribution data combines species observations with expert knowledge to derive range limits, and provides extent of occurrence (i.e., equal species occurrence across a polygon) rather than probability of occurrence, which is used in alternative datasets such as AquaMaps (*79*). We applied successive filtering steps to derive the final species list, which is detailed as follows. Using the R packages *rredlist* and *iucnredlist*, we extracted all IUCN species assessments for Vulnerable, Endangered, and Critically Endangered to acquire a list of marine species (*80*, *81*). We selected only marine species that fit within these Red List categories in a global assessment and excluded species that only existed as a population assessment. We then filtered our IUCN species list to only those that exist within the marine trait-based vulnerability framework (*47*). This resulted in a total of 1382 marine species (598 Vulnerable, 579 Endangered, and 205 Critically Endangered species) that have vulnerability values for our four anthropogenic stressors and exist within the three Red List categories.

We further filtered species based on the availability of species distribution data in the IUCN Red List (spatial data version update 27 March 2025) (*78*). We removed duplicate entries, aggregated split species entries, and aggregated subspecies entries into single species layers (e.g., *Balaenoptera musculus*). We also subset the species distribution to only those with IUCN presence values of extant, probably extant, or possibly extant. The IUCN species distribution data were transformed from their original World Geodetic System 1984 (ESRI:4326) coordinate reference system to our analysis grid with a resolution of 50 km by 50 km in the Mollweide (ESRI: 54009) coordinate reference system.

We filtered the species list to only those with available species distribution data resulting in a final list of 773 species for our analysis (Vulnerable: 361, Endangered: 280: Critically Endangered: 132).

### Trait-based vulnerability framework

We used vulnerability values developed from a global, trait-based marine vulnerability framework for each of our 773 species in relation to four anthropogenic stressors (*47*). This framework quantified species vulnerability for >44,000 marine species across 22 stressors, using 42 traits from five categories: biophysical traits, movement, reproduction, spatial scale, and specialization. We extracted vulnerability values for each of our species and matched them to the relevant stressors (see below) as follows: biomass removal was used for targeted fishing, bycatch for bycatch, wildlife strike for shipping, and habitat loss/degradation for habitat loss or degradation. For the impact calculation, each vulnerability value was applied to all grid cells where the species was present, producing four vulnerability rasters per species, each representing a uniform vulnerability to a stressor across its distribution.

### Stressor layers

Distribution maps of the intensity of the four stressors included in this study (i.e., a spatially explicit map showing the relative intensity of a stressor) were from Halpern *et al.* (*2*) and O’Hara *et al.* (*6*). Anthropogenic stressor distributions were provided as gridded data, with values ranging from 0 (stressor absent) to 1 (maximum intensity based on a Reference point). Stressor data were aggregated by averaging from their original 10 km by 10 km grid to the 50 km by 50 km analysis grid, both in the Mollweide (ESRI: 54009) coordinate reference system. For shipping, a single global map of relative stressor intensity was applied to all species. The habitat loss or degradation layer was created by applying min-max normalization after summing three individual layers: direct human disturbance along coastlines, habitat destruction from demersal destructive fishing, and degradation from benthic structures. This composite layer retained the 0-to-1 scale and was used for all species. The remaining two stressors, targeted fishing and bycatch, were dependent on species-specific information. The targeted fishing layer represents catch that is directly targeting that species and the bycatch layer is dependent on the water column position of a species (i.e., benthic, pelagic, or benthopelagic). For these stressors, species-specific stressor layers were used for each species. See O’Hara *et al.* (*6*) for details on the data sources, transformation, and reference point used for each stressor layer.

### Cumulative human impacts

Human impact on a species was calculated using the trait-based vulnerability values, following the methods described in O’Hara *et al.* (*6*) and Fleury *et al.* (*58*). The impact $I_{i}^{j}$ on species *i* of stressor *j* in a grid cell as the product of the stressor intensity $S_{i}$ and vulnerability $V_{ij}$ of species *i* to that stressor *j* is

$$I_{i}^{j}=V_{\mathrm{ij}}S_{i}$$ (1)

Cumulative *cml* human impact $I_{i}^{\mathrm{cml}}$ on species *i* in a grid cell was calculated by summing the impacts of all four stressors

$$I_{i}^{\mathrm{cml}}=\sum_{j=1}^{4}V_{\mathrm{ij}}S_{i}$$ (2)

This additive model does not account for potential synergistic or antagonistic interactions among stressors. A high impact score (i.e., value of 1) means that a species is both vulnerable to the stressor and the stressor exists at a high intensity. All parameters are unitless, and therefore, the resulting cumulative human impact values are also unitless.

The impact calculations generated 3092 (773 species × 4 stressors) raster layers, each representing the impact of a single stressor on a single species. The cumulative impact calculation is the sum of all four impact layers for each species generating 773 raster layers.

### Prioritization scenarios

We used the decision-support tool *prioritizr*, an R package, which compiles mixed integer linear programming problems and solves them using the Gurobi v12.0.1 optimizer (*57*, *82*). We chose *prioritizr* because integer linear programming approaches have been shown to outperform simulated annealing approaches commonly used in other conservation planning tools (*57*, *83*). *Prioritizr* offers a range of customizable functions, such as penalties, targets, and constraints, for configuring conservation problems, which enabled us to test multiple scenarios for incorporating human impact into spatial prioritization. The framework presented here is not restricted to *prioritizr* and could be implemented using other conservation planning tools that are valuable for optimizing cost-benefit trade-offs, such as Marxan (*84*); however, solution efficiency may vary depending on the tool used (*83*). Full configurations of each prioritization scenario are shown in Table 1.

The global ocean planning region for the spatial prioritizations had 145,894 PUs from which the prioritization solver may select or not select for protection based on the objective and features for each planning problem. We used a Mollweide coordinate reference system with a resolution of 50 km by 50 km for each planning problem. Each scenario used the minimum set objective to minimize total cost while ensuring representation targets are met for all features. We set species representation targets to reflect the representativeness component of the Global Biodiversity Framework Target 3, which not only establishes an overall 30% area-based conservation target but also calls for an ecologically representative system of protected areas (*7*). Spatial prioritization studies commonly operationalize this representativeness requirement using targets for species’ distributions or bioregions (*30*, *85*–*89*).

In our study, the minimum set objective was chosen as it is better suited for fully achieving conservation goals and allowed for comparisons across methods without limiting the costs (*57*). To maintain comparability between scenarios and demonstrate the outcome of the framework, additional parameters (e.g., boundary length modifiers or species penalty factors) were not included in the planning problem. Further, we used *prioritizr*, which uses integer linear programming to find the optimal solution rather than a local maximum as in Marxan, and therefore are comparing exact solutions not a single iteration or a traditional irreplaceability index (*57*). The resulting planning problem for each scenario strictly used the minimum set objective with the scenario-specific features and targets described in the following sections.

The prioritization solutions were compared by overlaying the solution from the reference scenario with either the Impact or Hybrid scenario. Each PU was then classified as selected by both scenarios, the Reference scenario, or either the Impact or Hybrid scenario. Percentages were calculated as the proportion of PUs in each class relative to the total number of PUs selected by at least one of the two scenarios in the comparison, excluding PUs not selected by either scenario.

### Cost layer

The cost layer used in our global prioritization analysis was based on the opportunity cost of fishing and follows the approach of Brito-Morales *et al.* (*90*). Briefly, we estimated the fisheries value (US$) of each PU from a global database of catch (kg) (*91*) and price (US$ kg^−1^) for each taxa caught (*92*). Catch and price data for 1314 taxa were successfully matched for 2008 to 2017. A total cost per PU (US$) was calculated by summing all taxa and includes an estimate of illegal, unreported, and unregulated (IUU) fishing and discards. We gap-filled any data gaps in the fisheries cost layers using a nearest-neighbor method with a 3 × 3 matrix. The majority of these data gaps existed within the Arctic and Southern Oceans having minimal effects on the overall cost layer.

### Reference scenario

The Reference scenario represents a common configuration in spatial conservation prioritization, where species distributions are used as features and conservation targets are set as a percentage of each species’ distribution. The Reference scenario includes 773 species distribution layers as features, with a target value of 0.3 (representative of 30% the area of the distribution of each species), as suggested by 2030 international commitment targets, applied across all species.

### Impact scenario

In the Impact scenario, only the 773 cumulative impact layers were used as features. The target for the cumulative impact layers was set to equal the total CIR percentage achieved in the Reference scenario (17.3%) and was applied across all species. This formulation ensures that impact reduction is equitable across all species, rather than being concentrated within a small subset.

### Hybrid scenario

In the Hybrid scenario, we build on the Reference scenario by incorporating cumulative human impact as an additional feature. Specifically, we used the 773 species distribution layers and the 773 cumulative impact layers, resulting in a total of 1546 features. Two separate target values were assigned: one for species distributions and another for cumulative impact. The species distribution target used a value of 0.3 across all species and the cumulative impact target was set to equal the calculated impact reduction percentage achieved in the Reference scenario (17.3%) and was applied across all species.

### Statistical software and data availability

All analyses were conducted in R Statistical Software v4.5.1 (*93*). Primary packages used include the following: *iucnredlist*, *prioritizr*, *rredlist*, *sf*, *spatialplanr*, *spatialgridr*, *terra*, and *tidyverse*.

## References

[1] E. Gomes, M. Inácio, K. Bogdzevič, M. Kalinauskas, D. Karnauskaitė, P. Pereira, Future land-use changes and its impacts on terrestrial ecosystem services: A review. Sci. Total Environ. 781, 146716 (2021). 10.1016/j.scitotenv.2021.146716 33798896

[2] B. S. Halpern, M. Frazier, J. Afflerbach, J. S. Lowndes, F. Micheli, C. O’Hara, C. Scarborough, K. A. Selkoe, Recent pace of change in human impact on the world’s ocean. Sci. Rep. 9, 11609 (2019). 10.1038/s41598-019-47201-9 PMC6691109 31406130

[3] B. S. Halpern, M. Frazier, C. C. O’Hara, O. A. Vargas-Fonseca, A. T. Lombard, Cumulative impacts to global marine ecosystems projected to more than double by mid-century. Science 389, 1216–1219 (2025). 10.1126/science.adv2906 40906781

[4] J.-B. Jouffray, R. Blasiak, A. V. Norström, H. Österblom, M. Nyström, The blue acceleration: The trajectory of human expansion into the ocean. One Earth 2, 43–54 (2020).

[5] G. Li, C. Fang, Y. Li, Z. Wang, S. Sun, S. He, W. Qi, C. Bao, H. Ma, Y. Fan, Y. Feng, X. Liu, Global impacts of future urban expansion on terrestrial vertebrate diversity. Nat. Commun. 13, 1628 (2022). 10.1038/s41467-022-29324-2 PMC8956596 35338145

[6] C. C. O’Hara, M. Frazier, M. Valle, N. Butt, K. Kaschner, C. Klein, B. S. Halpern, Cumulative human impacts on global marine fauna highlight risk to biological and functional diversity. PLOS ONE 19, e0309788 (2024). 10.1371/journal.pone.0309788 PMC11410257 39292645

[7] CBD, Kunming-Montreal Global Biodiversity Framework (CBD/COP/DEC/15/4, Convention on Biological Diversity, Montreal, Canada, 2022).

[8] UNGA, The UN Sustainable Development Goals (UNGA, New York, 2015); https://digitallibrary.un.org/record/800852.

[9] M. Maestro, M. L. Pérez-Cayeiro, J. A. Chica-Ruiz, H. Reyes, Marine protected areas in the 21st century: Current situation and trends. Ocean Coast. Manag. 171, 28–36 (2019).

[10] J. E. M. Watson, N. Dudley, D. B. Segan, M. Hockings, The performance and potential of protected areas. Nature 515, 67–73 (2014). 10.1038/nature13947 25373676

[11] K. R. Jones, C. J. Klein, B. S. Halpern, O. Venter, H. Grantham, C. D. Kuempel, N. Shumway, A. M. Friedlander, H. P. Possingham, J. E. M. Watson, The location and protection status of Earth’s diminishing marine wilderness. Curr. Biol. 28, 2506–2512.e3 (2018). 10.1016/j.cub.2018.06.010 30057308

[12] C. J. Klein, K. A. Wilson, M. Watts, J. Stein, J. Carwardine, B. Mackey, H. P. Possingham, Spatial conservation prioritization inclusive of wilderness quality: A case study of Australia’s biodiversity. Biol. Conserv. 142, 1282–1290 (2009).

[13] J. E. M. Watson, R. A. Fuller, A. W. T. Watson, B. G. Mackey, K. A. Wilson, H. S. Grantham, M. Turner, C. J. Klein, J. Carwardine, L. N. Joseph, H. P. Possingham, Wilderness and future conservation priorities in Australia. Divers. Distrib. 15, 1028–1036 (2009).

[14] D. C. Dunn, A.-L. Harrison, C. Curtice, S. DeLand, B. Donnelly, E. Fujioka, E. Heywood, C. Y. Kot, S. Poulin, M. Whitten, S. Åkesson, A. Alberini, W. Appeltans, J. M. Arcos, H. Bailey, L. T. Ballance, B. Block, H. Blondin, A. M. Boustany, J. Brenner, P. Catry, D. Cejudo, J. Cleary, P. Corkeron, D. P. Costa, M. Coyne, G. O. Crespo, T. E. Davies, M. P. Dias, F. Douvere, F. Ferretti, A. Formia, D. Freestone, A. S. Friedlaender, H. Frisch-Nwakanma, C. B. Froján, K. M. Gjerde, L. Glowka, B. J. Godley, J. Gonzalez-Solis, J. P. Granadeiro, V. Gunn, Y. Hashimoto, L. M. Hawkes, G. C. Hays, C. Hazin, J. Jimenez, D. E. Johnson, P. Luschi, S. M. Maxwell, C. McClellan, M. Modest, G. N. di Sciara, A. H. Palacio, D. M. Palacios, A. Pauly, M. Rayner, A. F. Rees, E. R. Salazar, D. Secor, A. M. M. Sequeira, M. Spalding, F. Spina, S. Van Parijs, B. Wallace, N. Varo-Cruz, M. Virtue, H. Weimerskirch, L. Wilson, B. Woodward, P. N. Halpin, The importance of migratory connectivity for global ocean policy. Proc. R. Soc. B 286, 20191472 (2019). 10.1098/rspb.2019.1472 PMC6784718 31551061

[15] K. C. V. Buenafe, D. C. Dunn, A. Metaxas, D. S. Schoeman, J. D. Everett, A. Pidd, J. O. Hanson, L. K. Bentley, S. W. Kim, S. Neubert, K. L. Scales, A. Dabalà, I. Brito-Morales, A. J. Richardson, Current approaches and future opportunities for climate-smart protected areas. Nat. Rev. Biodivers. 1, 284–297 (2025).

[16] L. M. Barr, R. L. Pressey, R. A. Fuller, D. B. Segan, E. McDonald-Madden, H. P. Possingham, A new way to measure the world’s protected area coverage. PLOS ONE 6, e24707 (2011). 10.1371/journal.pone.0024707 PMC3177831 21957458

[17] R. Devillers, R. L. Pressey, A. Grech, J. N. Kittinger, G. J. Edgar, T. Ward, R. Watson, Reinventing residual reserves in the sea: Are we favouring ease of establishment over need for protection? Aquat. Conserv. Mar. Freshw. Ecosyst. 25, 480–504 (2015).

[18] R. Devillers, R. L. Pressey, T. J. Ward, A. Grech, J. N. Kittinger, G. J. Edgar, R. A. Watson, Residual marine protected areas five years on: Are we still favouring ease of establishment over need for protection? Aquat. Conserv. Mar. Freshw. Ecosyst. 30, 1758–1764 (2020).

[19] K. Jantke, K. R. Jones, J. R. Allan, A. L. M. Chauvenet, J. E. M. Watson, H. P. Possingham, Poor ecological representation by an expensive reserve system: Evaluating 35 years of marine protected area expansion. Conserv. Lett. 11, e12584 (2018).

[20] L. N. Joppa, A. Pfaff, High and far: Biases in the location of protected areas. PLOS ONE 4, e8273 (2009). 10.1371/journal.pone.0008273 PMC2788247 20011603

[21] R. L. Pressey, P. Visconti, P. J. Ferraro, Making parks make a difference: Poor alignment of policy, planning and management with protected-area impact, and ways forward. Phil. Trans. R. Soc. B 370, 20140280 (2015). 10.1098/rstb.2014.0280 PMC4614736 26460132

[22] R. L. Pressey, P. Visconti, M. C. McKinnon, G. G. Gurney, M. D. Barnes, L. Glew, M. Maron, The mismeasure of conservation. Trends Ecol. Evol. 36, 808–821 (2021). 10.1016/j.tree.2021.06.008 34303527

[23] K. E. Roberts, G. A. Duffy, C. N. Cook, Bio-physical models of marine environments reveal biases in the representation of protected areas. Aquat. Conserv. Mar. Freshw. Ecosyst. 29, 499–510 (2019).

[24] E. Sala, J. Lubchenco, K. Grorud-Colvert, C. Novelli, C. Roberts, U. R. Sumaila, Assessing real progress towards effective ocean protection. Mar. Policy 91, 11–13 (2018).

[25] O. Venter, A. Magrach, N. Outram, C. J. Klein, H. P. Possingham, M. Di Marco, J. E. M. Watson, Bias in protected-area location and its effects on long-term aspirations of biodiversity conventions. Conserv. Biol. 32, 127–134 (2018). 10.1111/cobi.12970 28639356

[26] R. R. S. Vieira, R. L. Pressey, R. Loyola, The residual nature of protected areas in Brazil. Biol. Conserv. 233, 152–161 (2019).

[27] E. T. Game, E. McDonald-Madden, M. L. Puotinen, H. P. Possingham, Should we protect the strong or the weak? Risk, resilience, and the selection of marine protected areas. Conserv. Biol. 22, 1619–1629 (2008). 10.1111/j.1523-1739.2008.01037.x 18759769

[28] M. Davey, J. E. M. Watson, Australia’s inadequate marine protection. Science 387, 586–587 (2025). 10.1126/science.adt7848 39913587

[29] S. D’agata, D. Mouillot, L. Wantiez, A. M. Friedlander, M. Kulbicki, L. Vigliola, Marine reserves lag behind wilderness in the conservation of key functional roles. Nat. Commun. 7, 12000 (2016). 10.1038/ncomms12000 PMC4931279 27354026

[30] K. R. Jones, C. J. Klein, H. S. Grantham, H. P. Possingham, B. S. Halpern, N. D. Burgess, S. H. M. Butchart, J. G. Robinson, N. Kingston, N. Bhola, J. E. M. Watson, Area requirements to safeguard Earth’s marine species. One Earth 2, 188–196 (2020).

[31] C. J. Klein, C. J. Brown, B. S. Halpern, D. B. Segan, J. McGowan, M. Beger, J. E. M. Watson, Shortfalls in the global protected area network at representing marine biodiversity. Sci. Rep. 5, 17539 (2015). 10.1038/srep17539 PMC4668359 26631984

[32] F. A. Ridley, S. P. Rushton, E. J. Hickinbotham, A. J. Suggitt, P. J. K. McGowan, L. Mair, Global mismatches between threat mapping research effort and the potential of threat abatement actions to reduce extinction risk. Conserv. Biol. 38, e14271 (2024). 10.1111/cobi.14271 38623873

[33] A. S. Kukkala, A. Moilanen, Core concepts of spatial prioritisation in systematic conservation planning. Biol. Rev. 88, 443–464 (2013). 10.1111/brv.12008 PMC3654170 23279291

[34] H. Tallis, J. Fargione, E. Game, R. McDonald, L. Baumgarten, N. Bhagabati, R. Cortez, B. Griscom, J. Higgins, C. M. Kennedy, J. Kiesecker, T. Kroeger, T. Leberer, J. McGowan, L. Mandle, Y. J. Masuda, S. A. Morrison, S. Palmer, R. Shirer, P. Shyamsundar, N. H. Wolff, H. P. Possingham, Prioritizing actions: Spatial action maps for conservation. Ann. N. Y. Acad. Sci. 1505, 118–141 (2021). 10.1111/nyas.14651 PMC9290997 34176148

[35] P. F. Smallhorn-West, R. L. Pressey, Why does conservation minimize opportunity costs? Conserv. Sci. Pract. 4, e12808 (2022).

[36] J. Carwardine, K. A. Wilson, M. Watts, A. Etter, C. J. Klein, H. P. Possingham, Avoiding costly conservation mistakes: The importance of defining actions and costs in spatial priority setting. PLOS ONE 3, e2586 (2008). 10.1371/journal.pone.0002586 PMC2440517 18596914

[37] J. M. Reimer, R. Devillers, B. Trouillet, N. C. Ban, T. Agardy, J. Claudet, Conservation ready marine spatial planning. Mar. Policy 153, 105655 (2023).

[38] B. Trouillet, Reinventing marine spatial planning: A critical review of initiatives worldwide. J. Environ. Pol. Plan. 22, 441–459 (2020).

[39] V. J. Tulloch, A. I. Tulloch, P. Visconti, B. S. Halpern, J. E. Watson, M. C. Evans, N. A. Auerbach, M. Barnes, M. Beger, I. Chadès, S. Giakoumi, E. McDonald-Madden, N. J. Murray, J. Ringma, H. P. Possingham, Why do we map threats? Linking threat mapping with actions to make better conservation decisions. Front. Ecol. Environ. 13, 91–99 (2015).

[40] J. Claudet, C. W. Osenberg, P. Domenici, F. Badalamenti, M. Milazzo, J. M. Falcón, I. Bertocci, L. Benedetti-Cecchi, J.-A. García-Charton, R. Goñi, J. A. Borg, A. Forcada, G. A. de Lucia, Á. Pérez-Ruzafa, P. Afonso, A. Brito, I. Guala, L. L. Diréach, P. Sanchez-Jerez, P. J. Somerfield, S. Planes, Marine reserves: Fish life history and ecological traits matter. Ecol. Appl. 20, 830–839 (2010). 10.1890/08-2131.1 20437967

[41] B. S. Halpern, The impact of marine reserves: Do reserves work and does reserve size matter? Ecol. Appl. 13, 117–137 (2003).

[42] G. Hoppit, K. Nurkse, I. Beleem, N. Cadoni, T. Crowe, M. Bekaert, L. Bongiorni, K. Dvorski, G. Everaert, F. Frau, S. Jernberg, A. Krvarić, A. Kõivupuu, N. Malovrazić, G. Marchessaux, M. J. Perschke, H. C. Petersen, C. O. Quintana, K. J. Raatikainen, G. Sará, M. Sicard, M. Stevens, R. Szava-Kovats, A. Vaher, A. V. Gerven, F. R. Barboza, Enhancing marine protected areas with effective ecological and environmental data integration. Ecol. Indic. 178, 114119 (2025).

[43] S. Lester, B. Halpern, K. Grorud-Colvert, J. Lubchenco, B. Ruttenberg, S. Gaines, S. Airamé, R. Warner, Biological effects within no-take marine reserves: A global synthesis. Mar. Ecol. Prog. Ser. 384, 33–46 (2009).

[44] L. S. Waechter, O. J. Luiz, F. Leprieur, M. G. Bender, Functional biogeography of marine vertebrates in Atlantic Ocean reefs. Divers. Distrib. 28, 1680–1693 (2022).

[45] D. J. Baird, M. N. Rubach, P. J. Van den Brink, Trait-based ecological risk assessment (TERA): The new frontier. Integr. Environ. Assess. Manag. 4, 2–3 (2008). 10.1897/ieam_2007-063.1 18260202

[46] R. V. Gallagher, N. Butt, A. J. R. Carthey, A. Tulloch, L. Bland, S. Clulow, T. Newsome, R. Y. Dudaniec, V. M. Adams, A guide to using species trait data in conservation. One Earth 4, 927–936 (2021).

[47] N. Butt, B. S. Halpern, C. C. O’Hara, A. L. Allcock, B. Polidoro, S. Sherman, M. Byrne, C. Birkeland, R. G. Dwyer, M. Frazier, B. K. Woodworth, C. P. Arango, M. J. Kingsford, V. Udyawer, P. Hutchings, E. Scanes, E. J. McClaren, S. M. Maxwell, G. Diaz-Pulido, E. Dugan, B. A. Simmons, A. S. Wenger, C. Linardich, C. J. Klein, A trait-based framework for assessing the vulnerability of marine species to human impacts. Ecosphere 13, e3919 (2022).

[48] W. B. Foden, S. H. M. Butchart, S. N. Stuart, J.-C. Vié, H. R. Akçakaya, A. Angulo, L. M. DeVantier, A. Gutsche, E. Turak, L. Cao, S. D. Donner, V. Katariya, R. Bernard, R. A. Holland, A. F. Hughes, S. E. O’Hanlon, S. T. Garnett, Ç. H. Şekercioğlu, G. M. Mace, Identifying the world’s most climate change vulnerable species: A systematic trait-based assessment of all birds, amphibians and corals. PLOS ONE 8, e65427 (2013). 10.1371/journal.pone.0065427 PMC3680427 23950785

[49] J. Cohen, A coefficient of agreement for nominal scales. Educ. Psychol. Meas. 20, 37–46 (1960).

[50] J. R. Landis, G. G. Koch, The measurement of observer agreement for categorical data. Biometrics 33, 159 (1977). 843571

[51] C. Gaebel, C. Baulcomb, D. E. Johnson, J. M. Roberts, Recognising stakeholder conflict and encouraging consensus of ‘science-based management’ approaches for marine biodiversity beyond national jurisdiction (BBNJ). Front. Mar. Sci. 7, 10.3389/fmars.2020.557546 (2020).

[52] P. J. S. Jones, Fishing industry and related perspectives on the issues raised by no-take marine protected area proposals. Mar. Policy 32, 749–758 (2008).

[53] P. Pechacek, G. Li, J. Li, W. Wang, X. Wu, J. Xu, Compensation payments for downsides generated by protected areas. Ambio 42, 90–99 (2013). 10.1007/s13280-012-0330-1 PMC3547464 22766753

[54] J. G. Rodrigues, S. Villasante, I. Sousa-Pinto, Exploring perceptions to improve the outcomes of a marine protected area. Ecol. Soc. 29, 18 (2024).

[55] W. B. Foden, B. E. Young, H. R. Akçakaya, R. A. Garcia, A. A. Hoffmann, B. A. Stein, C. D. Thomas, C. J. Wheatley, D. Bickford, J. A. Carr, D. G. Hole, T. G. Martin, M. Pacifici, J. W. Pearce-Higgins, P. J. Platts, P. Visconti, J. E. M. Watson, B. Huntley, Climate change vulnerability assessment of species. WIREs Clim. Change 10, e551 (2019).

[56] M. Pacifici, W. B. Foden, P. Visconti, J. E. M. Watson, S. H. M. Butchart, K. M. Kovacs, B. R. Scheffers, D. G. Hole, T. G. Martin, H. R. Akçakaya, R. T. Corlett, B. Huntley, D. Bickford, J. A. Carr, A. A. Hoffmann, G. F. Midgley, P. Pearce-Kelly, R. G. Pearson, S. E. Williams, S. G. Willis, B. Young, C. Rondinini, Assessing species vulnerability to climate change. Nat. Clim. Change 5, 215–224 (2015).

[57] J. O. Hanson, R. Schuster, M. Strimas-Mackey, N. Morrell, B. P. M. Edwards, P. Arcese, J. R. Bennett, H. P. Possingham, Systematic conservation prioritization with the prioritizr R package. Conserv. Biol. 39, e14376 (2025). 10.1111/cobi.14376 PMC11780203 39268847

[58] A. G. Fleury, C. C. O’Hara, N. Butt, J. Restrepo, B. S. Halpern, C. J. Klein, C. D. Kuempel, K. M. Gaynor, L. K. Bentley, A. J. Richardson, D. C. Dunn, Spatial and life history variation in a trait-based species vulnerability and impact model. PLOS ONE 19, e0305950 (2024). 10.1371/journal.pone.0305950 PMC11192397 38905300

[59] E. E. Hodgson, T. E. Essington, I. C. Kaplan, Extending vulnerability assessment to include life stages considerations. PLOS ONE 11, e0158917 (2016). 10.1371/journal.pone.0158917 PMC4945077 27416031

[60] C. J. Klein, J. Beher, M. Chaloupka, M. Hamann, C. Limpus, H. P. Possingham, Prioritization of marine turtle management projects: A protocol that accounts for threats to different life history stages. Conserv. Lett. 10, 547–554 (2017).

[61] J. Restrepo, D. Nisthar, H. W.-K. Heng, L. K. Bentley, C. Curtice, S. DeLand, E. Fujioka, P. N. Halpin, S. K. Poulin, A. J. Richardson, J. A. Seminoff, R. A. Valverde, D. C. Dunn, Ecological insights and management implications of the global migratory connectivity of green turtles. bioRxiv 674769 [Preprint] (2025). 10.1101/2025.09.07.674769.

[62] B. S. Halpern, M. Frazier, J. Potapenko, K. S. Casey, K. Koenig, C. Longo, J. S. Lowndes, R. C. Rockwood, E. R. Selig, K. A. Selkoe, S. Walbridge, Spatial and temporal changes in cumulative human impacts on the world’s ocean. Nat. Commun. 6, 7615 (2015). 10.1038/ncomms8615 PMC4510691 26172980

[63] B. A. Nhancale, R. J. Smith, The influence of planning unit characteristics on the efficiency and spatial pattern of systematic conservation planning assessments. Biodivers. Conserv. 20, 1821–1835 (2011).

[64] G. W. Allison, J. Lubchenco, M. H. Carr, Marine reserves are necessary but not sufficient for marine conservation. Ecol. Appl. 8, S79–S92 (1998).

[65] E. Liz, A. Ruiz-Herrera, Potential impact of carry-over effects in the dynamics and management of seasonal populations. PLOS ONE 11, e0155579 (2016). 10.1371/journal.pone.0155579 PMC4865231 27171267

[66] C. M. O’Connor, S. J. Cooke, Ecological carryover effects complicate conservation. Ambio 44, 582–591 (2015). 10.1007/s13280-015-0630-3 PMC4552714 25678024

[67] C. M. Roberts, B. C. O’Leary, D. J. McCauley, P. M. Cury, C. M. Duarte, J. Lubchenco, D. Pauly, A. Sáenz-Arroyo, U. R. Sumaila, R. W. Wilson, B. Worm, J. C. Castilla, Marine reserves can mitigate and promote adaptation to climate change. Proc. Natl. Acad. Sci. U.S.A. 114, 6167–6175 (2017). 10.1073/pnas.1701262114 PMC5474809 28584096

[68] R. L. Pressey, R. Weeks, G. G. Gurney, From displacement activities to evidence-informed decisions in conservation. Biol. Conserv. 212, 337–348 (2017).

[69] G. McDonald, J. Bone, C. Costello, G. Englander, J. Raynor, Global expansion of marine protected areas and the redistribution of fishing effort. Proc. Natl. Acad. Sci. U.S.A. 121, e2400592121 (2024). 10.1073/pnas.2400592121 PMC11260147 38980905

[70] M. Bode, A. I. T. Tulloch, M. Mills, O. Venter, A. W. Ando, A conservation planning approach to mitigate the impacts of leakage from protected area networks. Conserv. Biol. 29, 765–774 (2015). 10.1111/cobi.12434 25494874

[71] P. Visconti, M. Bakkenes, R. J. Smith, L. Joppa, R. E. Sykes, Socio-economic and ecological impacts of global protected area expansion plans. Phil. Trans. R. Soc. B 370, 20140284 (2015). 10.1098/rstb.2014.0284 PMC4614740 26460136

[72] A. Borja, M. Elliott, H. Teixeira, V. Stelzenmüller, S. Katsanevakis, M. Coll, I. Galparsoro, S. Fraschetti, N. Papadopoulou, C. Lynam, T. Berg, J. H. Andersen, J. Carstensen, M. C. Leal, M. C. Uyarra, Addressing the cumulative impacts of multiple human pressures in marine systems, for the sustainable use of the seas. Front. Ocean Sustain. 1, 10.3389/focsu.2023.1308125 (2024).

[73] G. J. Edgar, R. D. Stuart-Smith, T. J. Willis, S. Kininmonth, S. C. Baker, S. Banks, N. S. Barrett, M. A. Becerro, A. T. F. Bernard, J. Berkhout, C. D. Buxton, S. J. Campbell, A. T. Cooper, M. Davey, S. C. Edgar, G. Försterra, D. E. Galván, A. J. Irigoyen, D. J. Kushner, R. Moura, P. E. Parnell, N. T. Shears, G. Soler, E. M. A. Strain, R. J. Thomson, Global conservation outcomes depend on marine protected areas with five key features. Nature 506, 216–220 (2014). 10.1038/nature13022 24499817

[74] D. A. Gill, M. B. Mascia, G. N. Ahmadia, L. Glew, S. E. Lester, M. Barnes, I. Craigie, E. S. Darling, C. M. Free, J. Geldmann, S. Holst, O. P. Jensen, A. T. White, X. Basurto, L. Coad, R. D. Gates, G. Guannel, P. J. Mumby, H. Thomas, S. Whitmee, S. Woodley, H. E. Fox, Capacity shortfalls hinder the performance of marine protected areas globally. Nature 543, 665–669 (2017). 10.1038/nature21708 28329771

[75] G. Cobb, J. Nalau, A. L. M. Chauvenet, Global trends in geospatial conservation planning: A review of priorities and missing dimensions. Front. Ecol. Evol. 11, 10.3389/fevo.2023.1209620 (2024).

[76] E. M. De Santo, Missing marine protected area (MPA) targets: How the push for quantity over quality undermines sustainability and social justice. J. Environ. Manage. 124, 137–146 (2013). 10.1016/j.jenvman.2013.01.033 23582739

[77] J. W. White, J. K. Hopf, N. Arafeh-Dalmau, N. C. Ban, A. E. Bates, J. Claudet, C. Lopazanski, J. M. Sunday, J. E. Caselle, Measurements, mechanisms, and management recommendations for how marine protected areas can provide climate resilience. Mar. Policy 171, 106419 (2025).

[78] IUCN, *The IUCN Red List of Threatened Species. Version 2025-1* (2025); https://iucnredlist.org/.

[79] C. C. O’Hara, J. C. Afflerbach, C. Scarborough, K. Kaschner, B. S. Halpern, Aligning marine species range data to better serve science and conservation. PLOS ONE 12, e0175739 (2017). 10.1371/journal.pone.0175739 PMC5414950 28467508

[80] W. Gearty, S. Chamberlain, Rredlist: “IUCN” Red List Client (2025); https://docs.ropensci.org/rredlist/.

[81] S. Tarr, I. Townsend, Iucnredlist: An R Package for the IUCN Red List API (2025); https://github.com/IUCN-UK/iucnredlist.

[82] Gurobi Optimization, LLC, Gurobi Optimizer Reference Manual version 12.0.1 (2025); https://gurobi.com.

[83] R. Schuster, J. O. Hanson, M. Strimas-Mackey, J. R. Bennett, Exact integer linear programming solvers outperform simulated annealing for solving conservation planning problems. PeerJ 8, e9258 (2020). 10.7717/peerj.9258 PMC7261139 32518737

[84] I. R. Ball, H. P. Possingham, M. E. Watts, “Marxan and relatives: Software for spatial conservation prioritization,” in *Spatial Conservation Prioritisation: Quantitative Methods and Computational Tools*, A. Moilanen, K. A. Wilson, H. P. Possingham, Eds. (Oxford University Press, 2009), pp. 185–210.

[85] M. E. Visalli, B. D. Best, R. B. Cabral, W. W. L. Cheung, N. A. Clark, C. Garilao, K. Kaschner, K. Kesner-Reyes, V. W. Y. Lam, S. M. Maxwell, J. Mayorga, H. V. Moeller, L. Morgan, G. O. Crespo, M. L. Pinsky, T. D. White, D. J. McCauley, Data-driven approach for highlighting priority areas for protection in marine areas beyond national jurisdiction. Mar. Policy 122, 103927 (2020).

[86] E. Boyse, S. J. Goodman, M. Beger, Spatial conservation priorities for marine megafaunal predators: Multi-taxon versus taxon-specific approaches. Conserv. Sci. Pract. 7, e70092 (2025).

[87] S. Karimi, R. Schuster, J. O. Hanson, F. Riva, A. Liczner, J. R. Bennett, Priority areas to conserve biodiversity in Canada. FACETS 10, 1–10 (2025).

[88] R. Schuster, S. Wilson, A. D. Rodewald, P. Arcese, D. Fink, T. Auer, J. R. Bennett, Optimizing the conservation of migratory species over their full annual cycle. Nat. Commun. 10, 1754 (2019). 10.1038/s41467-019-09723-8 PMC6465267 30988288

[89] E. Hammill, A. I. T. Tulloch, H. P. Possingham, N. Strange, K. A. Wilson, Factoring attitudes towards armed conflict risk into selection of protected areas for conservation. Nat. Commun. 7, 11042 (2016). 10.1038/ncomms11042 PMC4820849 27025894

[90] I. Brito-Morales, D. S. Schoeman, J. D. Everett, C. J. Klein, D. C. Dunn, J. García Molinos, M. T. Burrows, K. C. V. Buenafe, R. M. Dominguez, H. P. Possingham, A. J. Richardson, Towards climate-smart, three-dimensional protected areas for biodiversity conservation in the high seas. Nat. Clim. Chang. 12, 402–407 (2022).

[91] R. A. Watson, A database of global marine commercial, small-scale, illegal and unreported fisheries catch 1950–2014. Sci. Data 4, 170039 (2017). 10.1038/sdata.2017.39 PMC5387926 28398351

[92] T. C. Tai, T. Cashion, V. W. Y. Lam, W. Swartz, U. R. Sumaila, Ex-vessel fish price database: Disaggregating prices for low-priced species from reduction fisheries. Front. Mar. Sci. 4, 10.3389/fmars.2017.00363 (2017).

[93] R Core Team, *R: A Language and Environment for Statistical Computing* (R Foundation for Statistical Computing, 2025); https://R-project.org/.

